# Reynolds nano fluid model for Casson fluid flow conveying exponential nanoparticles through a slandering sheet

**DOI:** 10.1038/s41598-023-28515-1

**Published:** 2023-02-02

**Authors:** Sohail Nadeem, Bushra Ishtiaq, Mohamed Bechir Ben Hamida, Shahah Almutairi, Hassan Ali Ghazwani, Sayed M. Eldin, A. S. Al-Shafay

**Affiliations:** 1 Department of Mathematics, Quaid-I-Azam University, 45320, Islamabad, 44000 Pakistan; 2 Department of Mathematics, Wenzhou University, Wenzhou, 325035 China; 3 Department of Mechanical Engineering, College of Engineering, Imam Mohammad Ibn Saud Islamic University (IMSIU), Riyadh, Saudi Arabia; 4 Research Laboratory of Ionized Backgrounds and Reagents Studies (EMIR), Preparatory Institute for Engineering Studies of Monastir (IPEIM), University of Monastir, Monastir City, Tunisia; 5 Department of Physics, Higher School of Sciences and Technology of Hammam Sousse (ESSTHS), University of Sousse, Sousse, Tunisia; 6 Mathematics Department, Faculty of Sciences, Northern Border University, Arar, 1321 Saudi Arabia; 7 Department of Mechanical Engineering, Faculty of Engineering, Jazan University, P.O box 45124, Jazan, Saudi Arabia; 8 Center of Research, Faculty of Engineering, Future University in Egypt, New Cairo, 11835 Egypt; 9 Department of Mechanical Engineering, College of Engineering, Prince Sattam Bin Abdulaziz University, Alkharj, Saudi Arabia

**Keywords:** Engineering, Mathematics and computing

## Abstract

Nanofluids with their augmented thermal characteristics exhibit numerous implementations in engineering and industrial fields such as heat exchangers, microelectronics, chiller, pharmaceutical procedures, etc. Due to such properties of nanofluids, a mathematical model of non-Newtonian Casson nanofluid is analyzed in this current study to explore the steady flow mechanism with the contribution of water-based Aluminum oxide nanoparticles. A stretchable surface incorporating variable thickness is considered to be the source of the concerning fluid flow in two-dimension. An exponential viscosity of the nanofluid is proposed to observe the fluid flow phenomenon. Different models of viscosity including Brinkman and Einstein are also incorporated in the flow analysis and compared with the present exponential model. The physical flow problem is organized in the boundary layer equations which are further tackled by the execution of the relevant similarity transformations and appear in the form of ordinary nonlinear differential equations. The different three models of nanofluid viscosity exhibit strong graphical and tabulated relations with each other relative to the various aspects of the flow problem. In all concerned models of the viscosity, the deteriorating nature of the velocity field corresponding to the Casson fluid and surface thickness parameters is observed.

## Introduction

The heat transfer mechanism has gained significant importance due to its performance in advanced industries, microelectronics, and chemical engineering. The thermal conductivity and rate of heat transfer are minimum in ordinary fluid (water, polymeric solution, oil, engine oils, ethylene glycol) which does not fulfill the requirement of modern necessities. To overcome this drawback of common fluids, Choi^1^ introduced the term nanofluid which is defined as the collaboration of nanoparticles into ordinary fluids. The heat transfer rate and thermal conductivity of an ordinary fluid can be augmented with the involvement of the nano-sized particle having a small concentration. After the development of the nanofluid, numerous researchers have devoted their attention to increasing the common fluid’s thermal conductivity through the addition of nanoparticles. Rawat et al.^2^ considered the water-based Copper nanoparticle in the formulation of nanofluid and investigate the time-independent flow over a cone with a porous surface. Through a vertical medium, the model-based study of nanofluid flows with the magnetic field was conducted by Reza-E-Rabbi et al.^3^. The laminar flow phenomenon generated by a permeable plate with the heat transfer mechanism in nanofluid was scrutinized by Maleki et al.^4^. Reza-E-Rabbi et al.^5^ considered a stretchable inclined surface to explore the time-independent flow mechanism in a nanofluid influenced by chemical reactions. Arabpour et al.^6^ conducted a study of nanofluid flow in a rectangular microchannel and inspect the mechanism of heat transfer. The hydromagnetic time-dependent flow behavior of a nanofluid comprising three different kinds of nanoparticles with traditional fluid water was investigated Das by et al.^7^ with entropy production. Disu and Dada^8^ assumed a porous channel to discuss the hydromagnetic flow in two dimensions by taking the viscosity model of an exponential form. Through dimensionless groups, Masoud Hosseini et al.^9^ worked on a model of nanofluid’s viscosity with exponential form and compared the obtained outcomes with the other models. Namburu et al.^10^ studied the flow of a nanofluid with the involvement of concentration and temperature in the exponential form of viscosity. At two different ranges of temperature, Sahoo et al.^11^ examined the thermophysical characteristics of a nanofluid with Aluminum oxide nanoparticles and proposed an exponential type of viscosity of the nanofluid. Recently many studies^12–16^ have been conducted on the flow phenomenon of nanofluids over various surfaces. Due to the complex rheology of non-Newtonian fluids, the shear stress and strain rate relationship cannot be discussed through a single constitutive equation. To define all the characteristics of non-Newtonian fluids, numerous models of such fluids have been introduced. Non-Newtonian fluids have been studied significantly because of the wide range of implementations in manufacturing procedures, industrial, and engineering fields such as drilling mud, polymer production, hot rolling, etc. Casson fluid^17^ belongs to the category of differential type non-Newtonian fluid due to its shear stress and strain concerning rheological characteristics. Beyond the critical value of stress, the behavior of Casson fluid becomes from non-Newtonian to Newtonian and at small shear strain, it displays the elastic solid performance. Various foodstuffs such as orange juice, chocolate, tomato sauce, syrups, human blood, and soup^18,19^ illustrate the features of the Casson fluid model. Many researchers briefly studied the flow behavior of Casson fluids in the few past years. With the involvement of a shrunk sheet, Haq et al.^20^ observed the flow mechanism of a Casson nanofluid with magnetic effects. The flow system of Casson nanofluid with magnetic field and heat transfer phenomenon with the impact of nonlinear thermal radiation was demonstrated by Ghadikolaei et al.^21^. They examined that the flow system velocity exhibits the descending behavior relative to the higher intensity of the Casson fluid parameter. The significance of the Buongiorno model on the radiative flow of a Casson fluid generated by a stretchable surface was investigated by Reza-E-Rabbi et al.^22^. Javed and Nadeem^23^ assumed two concentric cylinders to explore the flow phenomenon of a Casson nanofluid and presented the outcomes numerically. An exploration of the chemical reaction and periodic magnetic field on the flow system of Casson nanofluid initiated by a stretchable permeable sheet was carried out by Al-Mamun et al.^24^. An exploration of nonlinear thermal radiation effects with Arrhenius activation energy on the flow phenomenon of Casson nanofluid was scrutinized by Reza-E-Rabbi et al.^25^. Liu et al.^26^ discuss the Casson nanofluid in a time-dependent flow system near a stagnant point above a stretched radiative sheet. With the involvement of an exponentially shrunk vertical surface, Ishtiaq and Nadeem^24^ scrutinized the flow mechanism with an inclined magnetic field and the Buongiorno model in a Casson nanofluid. Al-Mamun et al.^27^ theoretically investigated the heat transfer mechanism and flow behavior of a Casson nanofluid with the significance of the periodic magnetic field.

The heat transfer mechanism and development of fluid flow due to a stretchable surface have gained significant importance with various applications such as the production of polymer sheets, glass blowing, paper construction, and metallic plate cooling. The flow of fluid above a nonlinear/linear, non-stretched/stretched surface has been researched by many scientists but the most interesting surface is the stretchable surface incorporating variable thickness (slendering surface). The real-life practices of such surfaces incorporate metallurgical procedures, plastic film, polymer extrusion, metal sparing, etc. Ahmad et al.^28^ worked on the slendering surface to explore the flow mechanism of a Maxwell nanofluid with various slip effects. The time-independent MHD incompressible flow phenomenon through a stagnant point generated by a slendering extended sheet in a micropolar fluid was scrutinized by Anantha et al.^29^. An estimation of the magnetic and slip impacts on the flow behavior of a viscous Casson fluid subject to a surface having inconsistent thickness was carried out by Akolade et al.^30^. The thermal radiative flow phenomenon of Williamson and Casson fluids with the magnetic field produced by a stretchable surface of variable thickness was demonstrated by Saravana et al.^31^. They had come up with the point that the Casson fluid has a higher rate of heat transfer in comparison to Williamson fluid. On a slandering surface, the flow mechanism of Casson nanofluid with graphene nanoparticles was studied by Durgaprasad et al.^32^.

The fluid flow due to the slendering surface has attained little attention. The current study focuses on the boundary layer two-dimensional steady flow of Casson nanofluid with water based ${\mathrm{Al}}_{2}{\mathrm{O}}_{3}$ nanoparticle of exponential form subject to a slandering stretchable sheet. The main focus of this study is on the thermophysical properties of the concerned nanofluid in which we examine a new model of nanofluid viscosity with exponential form. No one utilize this model on the flow mechanism of Casson nanofluid until now. Furthermore, the viscosity models of Brinkman and Einstein are also involved. By considering the Einstein model, Brinkman model, and exponential form of nanofluid viscosity, we scrutinize the various aspects of the flow system of considered Casson nanofluid. For three concerned models of viscosity, the consequences of the physical parameters on the temperature distribution and flow velocity are briefly explained. The promising physical quantities of surface drag force and heat transfer rate are evaluated and compared in all cases of the nanofluid viscosity.

## Formulation of the problem

**Figure 1:**
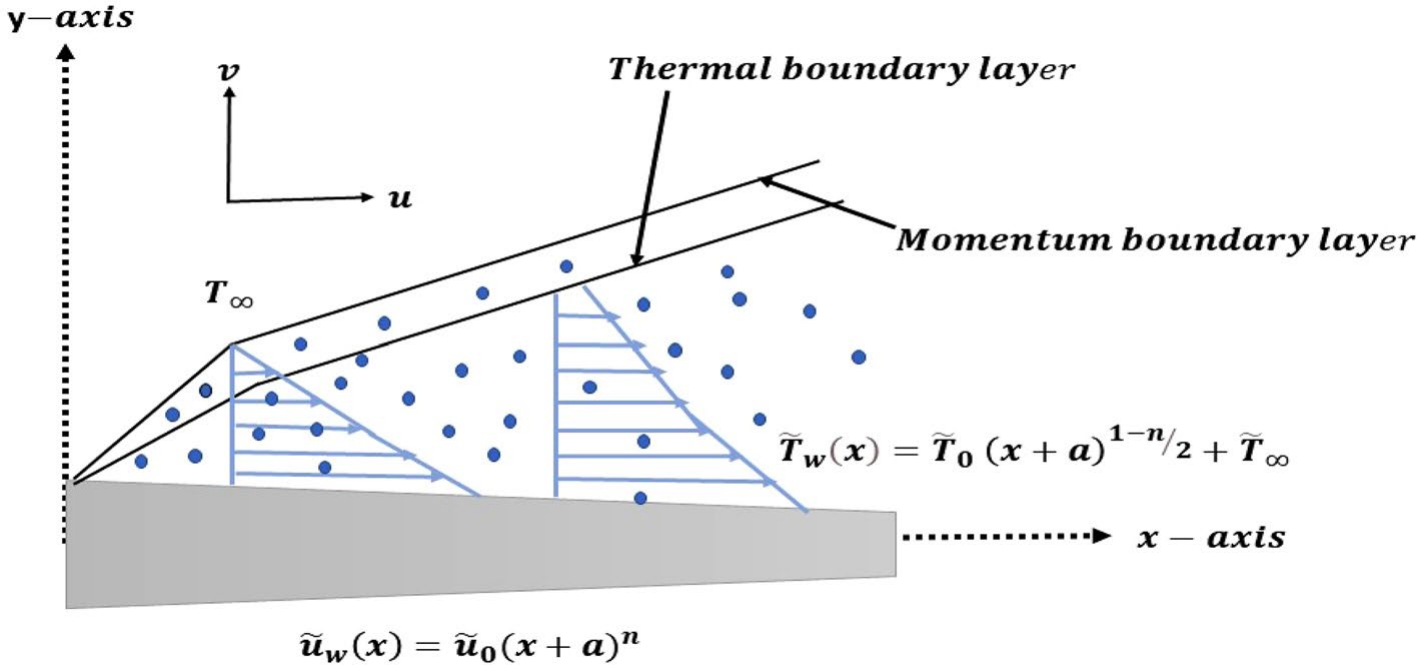
Geometry of the flow problem.

To formulate the concerned nanofluid ${\mathrm{Al}}_{2}{\mathrm{O}}_{3}-\mathrm{water}$, we consider the combination of Aluminum Oxide nanoparticle with water as the base fluid. In the Cartesian coordinate setup, a stretchable surface of variable thickness $y=J{(x+a)}^{(1-n)/2}, n\ne 1$ is situated in the x-direction while the y-axis is located perpendicular to the slendering surface for the incompressible flow problem of Casson nanofluid (See Fig. 1). The considered sheet is assumed to be stretched nonlinearly with ${\widetilde{u}}_{w}\left(x\right)={\widetilde{u}}_{0}{(x+a)}^{n}$ velocity.

The governing equations of the time-independent two-dimensional flow mechanism are exhibited as follows^33^

1 $$\frac{\partial \widetilde{u}}{\partial x}+\frac{\partial \widetilde{v}}{\partial y}=0,$$

2 $$\widetilde{u}\frac{\partial \widetilde{u}}{\partial x}+\widetilde{v}\frac{\partial \widetilde{v}}{\partial y}=\frac{{\mu }_{n\widetilde{f}}}{{\rho }_{n\widetilde{f}}}\left(1+{\beta }^{-1}\right)\frac{{\partial }^{2}\widetilde{u}}{\partial {y}^{2}},$$

3 $$\widetilde{u}\frac{\partial \widetilde{T}}{\partial x}+\widetilde{v}\frac{\partial \widetilde{T}}{\partial y}=\frac{{k}_{n\widetilde{f}}}{{\left({\rho c}_{p}\right)}_{n\widetilde{f}}}\frac{{\partial }^{2}\widetilde{T}}{\partial {y}^{2}},$$

The above system of Eqs. (1)–(3) has the following relevant boundary conditions^34^

4 $$\begin{gathered}\widetilde{u}\left(x,y\right)={\widetilde{u}}_{w}\left(x\right), \quad \widetilde{v}\left(x,y\right)=0, \quad \widetilde{T}\left(x,y\right)={\widetilde{T}}_{w}\left(x\right) \,\mathrm{at}\, y=J{(x+a)}^{(1-n)/2}\\ \widetilde{u}\left(x,y\right)=0, \quad \widetilde{T}\left(x,y\right)={\widetilde{T}}_{\infty } \,\mathrm{at}\, y=\infty .\end{gathered}$$

where

5 $${\widetilde{u}}_{w}\left(x\right)={\widetilde{u}}_{0}{(x+a)}^{n}, \quad {\widetilde{T}}_{w}\left(x\right)={\widetilde{T}}_{0} {(x+a)}^{(1-n)/2}+{\widetilde{T}}_{\infty }, \quad n\ne 1.$$

Now, the nonlinear coupled ODEs are acquired with the aid of the following similarity transformations^34^

6 $$\begin{gathered} \psi \left(x,y\right)=\sqrt{{\upsilon }_{\widetilde{f}}{\widetilde{u}}_{0}\left(\frac{2}{n+1}\right){\left(x+a\right)}^{n+1}}\widetilde{f}\left(\zeta \right), \quad \zeta =y\sqrt{{\widetilde{u}}_{0}\left(\frac{n+1}{2}\right)\left(\frac{{\left(x+a\right)}^{n-1}}{{\upsilon }_{\widetilde{f}}}\right)}\\ \widetilde{u}={\widetilde{u}}_{0}{\left(x+a\right)}^{n}{\widetilde{f}}^{^{\prime}}\left(\zeta \right), \quad \widetilde{v}=-\sqrt{\frac{n+1}{2}{\upsilon }_{\widetilde{f}}{\widetilde{u}}_{0}{\left(x+a\right)}^{n-1}}\left[{\widetilde{f}}^{^{\prime}}\left(\zeta \right)\left(\frac{n-1}{n+1}\right)+f(\zeta )\right]\\ \widetilde{\theta }\left(\zeta \right)=\frac{\widetilde{T}-{\widetilde{T}}_{\infty }}{{\widetilde{T}}_{w}\left(x\right)-{\widetilde{T}}_{\infty }}\end{gathered}.$$

The implementation of Eq. (6) to Eqs. (2) and (3) yield the following setup of equations

7 $$\left(\frac{{\mu }_{n\widetilde{f}}/{\mu }_{\widetilde{f}}}{{\rho }_{n\widetilde{f}}/{\rho }_{\widetilde{f}}}\right)\left(1+\frac{1}{\beta }\right){\widetilde{f}}^{{{\prime\prime\prime}}}+\widetilde{f}{\widetilde{f}}^{{{\prime\prime}}}-\frac{2n}{n+1}{\widetilde{{f}{^{\prime}}}}^{2}=0$$

8 $$\left( {\frac{{k_{{n\widetilde{f}}} /k_{{\widetilde{f}}} }}{{\left( {\rho c_{p} } \right)_{{n\widetilde{f}}} /\left( {\rho c_{p} } \right)_{{\widetilde{f}}} }}} \right)\widetilde{\theta }^{{\prime \prime }} - \Pr \left( {\frac{{1 - n}}{{n + 1}}\widetilde{f}^{\prime } \theta - \widetilde{f}\widetilde{\theta }^{\prime } } \right) = 0.$$

Equations (4) and (5) implies that

9 $$\begin{aligned}&\widetilde{f}\left(\delta \right)=\delta \left(\frac{1-n}{1+n}\right), \quad {\widetilde{f}}^{^{\prime}}\left(\delta \right)=1, \quad {\widetilde{f}}^{^{\prime}}\left(\infty \right)=0\\ & \widetilde{\theta }\left(\delta \right)=1, \quad \widetilde{\theta }\left(\infty \right)=0 , \quad n\ne 1\end{aligned}$$

Here

10 $$Pr={\mu }_{\widetilde{f}}{{c}_{p}}_{\widetilde{f}}/{k}_{\widetilde{f}}, \quad \delta =J\sqrt{{\widetilde{u}}_{0}(1+n)/2{\upsilon }_{\widetilde{f}}}.$$

The domain of the nonlinear Eqs. (7)–(9) is $[\delta ,\infty )$. Thus, to transform the domain from $[\delta ,\infty )$ into $[0,\infty )$, we describe^34^

11 $$F\left(\xi \right)=F\left(\zeta -\delta \right)=\widetilde{f}\left(\zeta \right), \quad \Theta \left(\xi \right)=\Theta \left(\zeta -\delta \right)=\widetilde{\theta }(\zeta ).$$

Now, the differential nonlinear Eqs. (7) and (8) with conditions (9) take the following form

12 $$\left(\frac{{\mu }_{n\widetilde{f}}/{\mu }_{\widetilde{f}}}{{\rho }_{n\widetilde{f}}/{\rho }_{\widetilde{f}}}\right)\left(1+\frac{1}{\beta }\right){F}^{{^{\prime}}{^{\prime}}{^{\prime}}}+FF{^{\prime}}{^{\prime}}-\frac{2n}{n+1}{F{^{\prime}}}^{2}=0$$

13 $$\left(\frac{{k}_{n\widetilde{f}}/{k}_{\widetilde{f}}}{{\left({\rho c}_{p}\right)}_{n\widetilde{f}}/{\left({\rho c}_{p}\right)}_{\widetilde{f}}}\right)\Theta {^{\prime}}{^{\prime}}-Pr\left(\frac{1-n}{n+1}F{^{\prime}}\Theta -F\Theta {^{\prime}}\right)=0$$

14 $$\begin{aligned}&F\left(0\right)=\delta \left(\frac{1-n}{1+n}\right), \quad F{^{\prime}}\left(0\right)=1, \quad F{^{\prime}}\left(\infty \right)=0\\ &\Theta \left(0\right)=1, \quad \Theta \left(\infty \right)=0 , \quad n\ne 1\end{aligned}$$

The Einstein model^35^ for the nanofluid viscosity has the following expression

15a $$\frac{{\mu }_{n\widetilde{f}}}{{\mu }_{\widetilde{f}}}=1+2.5\phi$$

The nanofluid viscosity in the Brinkman model^36^ is defined as

15b $$\frac{{\mu }_{n\widetilde{f}}}{{\mu }_{\widetilde{f}}}=\frac{1}{{(1-\phi )}^{5/2}}$$

In the ongoing flow problem, we take the exponential nanoparticle with viscosity in the following form

16a $$\frac{{\mu }_{n\widetilde{f}}}{{\mu }_{\widetilde{f}}}=\mathrm{Exp}[A\phi ]= 1+A\phi+\cdots$$

In (16a), if we take $A=2.5$ then we have the viscosity model of Einstein and Brinkman. Similarly, the expression for hybrid nanofluid can be defined as

16b $$\frac{{\mu }_{n\widetilde{f}}}{{\mu }_{\widetilde{f}}}=\mathrm{Exp}\left[M{\phi }_{1}+N{\phi }_{2}\right],$$

where $M$ and $N$ are constants.

The other properties of the nanofluid are mathematically expressed as^13^

17 $$\begin{gathered}\frac{{\left({\rho c}_{p}\right)}_{n\widetilde{f}}}{{\left({\rho c}_{p}\right)}_{\widetilde{f}}}=1+\phi \frac{{\left({\rho c}_{p}\right)}_{\widetilde{s}}}{{\left({\rho c}_{p}\right)}_{\widetilde{f}}}-\phi , \quad \frac{{\rho }_{n\widetilde{f}}}{{\rho }_{\widetilde{f}}}=1+\phi \frac{{\rho }_{\widetilde{s}}}{{\rho }_{\widetilde{f}}}-\phi , \\ \frac{{k}_{n\widetilde{f}}}{{k}_{\widetilde{f}}}=\frac{{k}_{\widetilde{s}}+2{k}_{\widetilde{f}}-2\phi ({k}_{\widetilde{f}}-{k}_{\widetilde{s}})}{{k}_{\widetilde{s}}+2{k}_{\widetilde{f}}+\phi ({k}_{\widetilde{f}}-{k}_{\widetilde{s}})}\end{gathered}.$$

The thermophysical characteristics of the concerned nanofluid with numerical values are depicted in Table 1.

**Table 1:** Thermophysical properties of Aluminum Oxide and base fluid water^37^.

Properties	Aluminum oxide ($${Al}_{2}{O}_{3})$$	Water $$({H}_{2}O)$$
${c}_{p} \left(J/kgK\right)$	765	4179
$k(1/{W}^{-1}mK)$	40	0.613
$\rho (kg{m}^{-3}$)	3970	997.1

The ongoing flow problem has promising physical quantities local Nusselt number and Skin friction coefficient. These quantities have the following expressions^25,26^

18 $$\begin{gathered}{C}_{f}=\frac{{\tau }_{w}}{1/2{\rho }_{\widetilde{f}}{\widetilde{u}}_{w}^{2}}, \quad {\tau }_{w}={\mu }_{n\widetilde{f}}(1+{\beta }^{-1}){\left(\frac{\partial \widetilde{u}}{\partial x}\right)}_{y=J{(x+a)}^{n/2}}\\ {Nu}_{x}=\frac{(x+a){q}_{w}}{{k}_{\widetilde{f}}{\widetilde{(T}}_{w}\left(x\right)-{\widetilde{T}}_{\infty })}, \quad {q}_{w}=-{k}_{n\widetilde{f}}{\left(\frac{\partial \widetilde{T}}{\partial y}\right)}_{y=J{(x+a)}^{n/2}}\end{gathered}.$$

Equations (6) and (18) yield the following expressions

19 $$\begin{gathered}{C}_{f}{{Re}_{x}}^\frac{1}{2}=2\frac{{\mu }_{n\widetilde{f}}}{{\mu }_{\widetilde{f}}}{\left(\frac{1+n}{2}\right)}^{1/2}(1+{\beta }^{-1})F{^{\prime}}{^{\prime}}(0),\\ {Nu}_{x}{{Re}_{x}}^{-\frac{1}{2}}=-\frac{{k}_{n\widetilde{f}}}{{k}_{\widetilde{f}}}{\left(\frac{1+n}{2}\right)}^{1/2}{\Theta }^{^{\prime}}\left(0\right), {Re}_{x}=\left(x+a\right){\widetilde{u}}_{w}/{\upsilon }_{\widetilde{f}} \end{gathered}.$$

## Results and discussion

**Figure 2:**
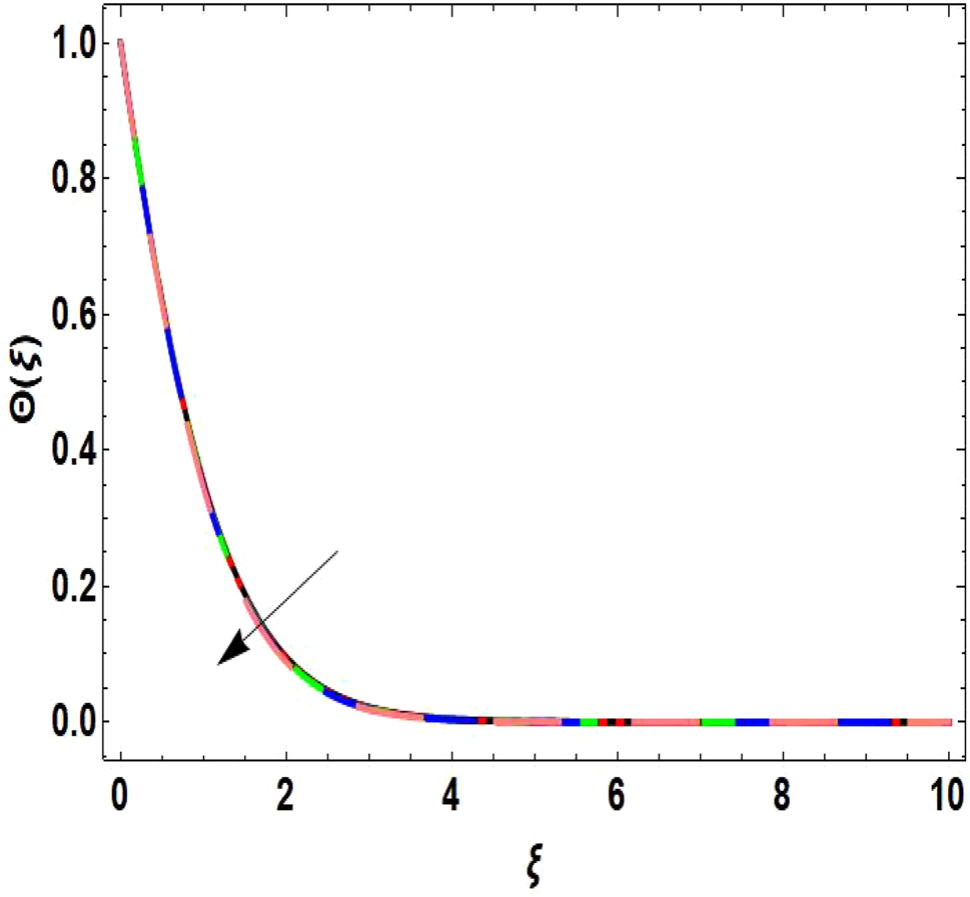
Temperature curve relative to exponential viscosity when $A=0.5, 2.5, 5.0, 7.0, 10.0.$

**Figure 3:**
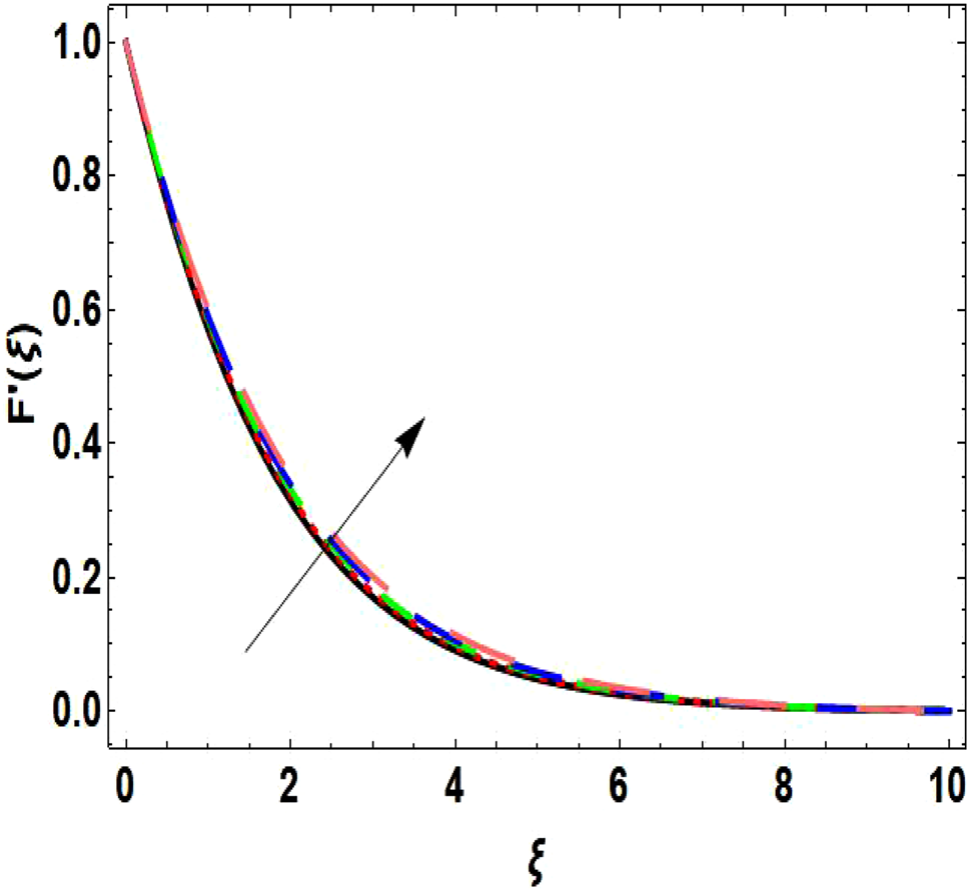
Velocity curve relative to exponential viscosity when $A=0.5, 2.5, 5.0, 7.0, 10.0.$

**Figure 4:**
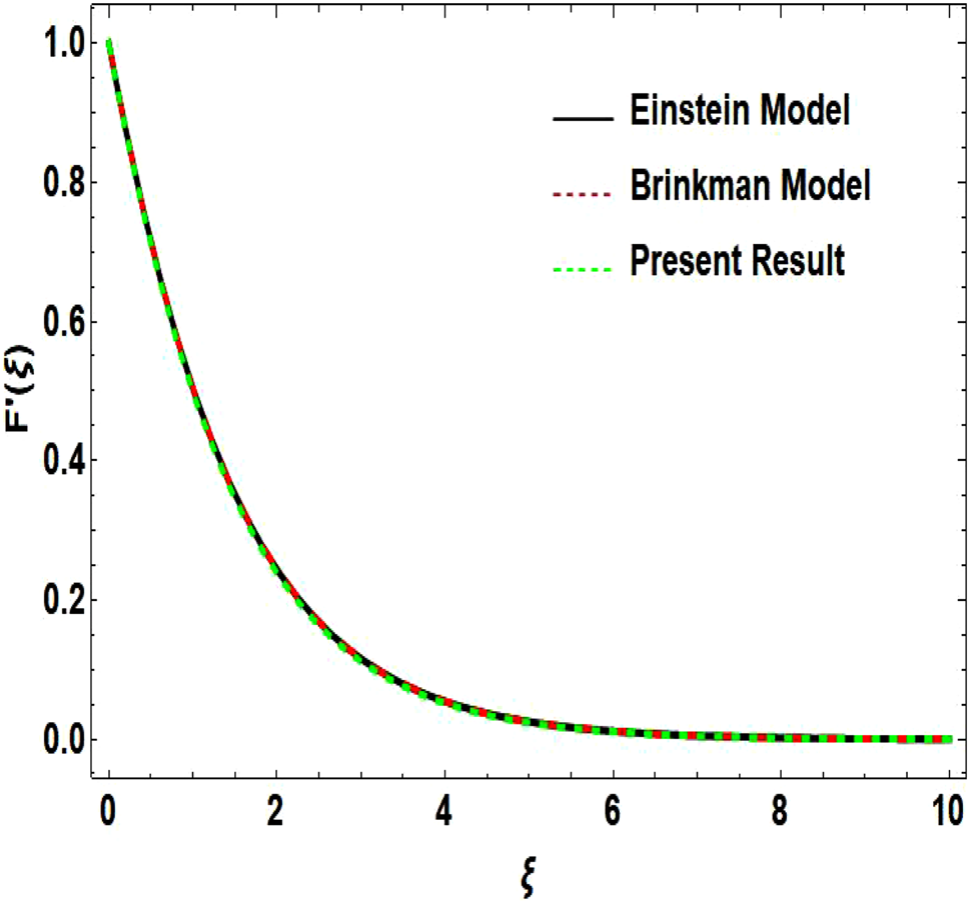
Velocity curve relative to different models.

**Figure 5:**
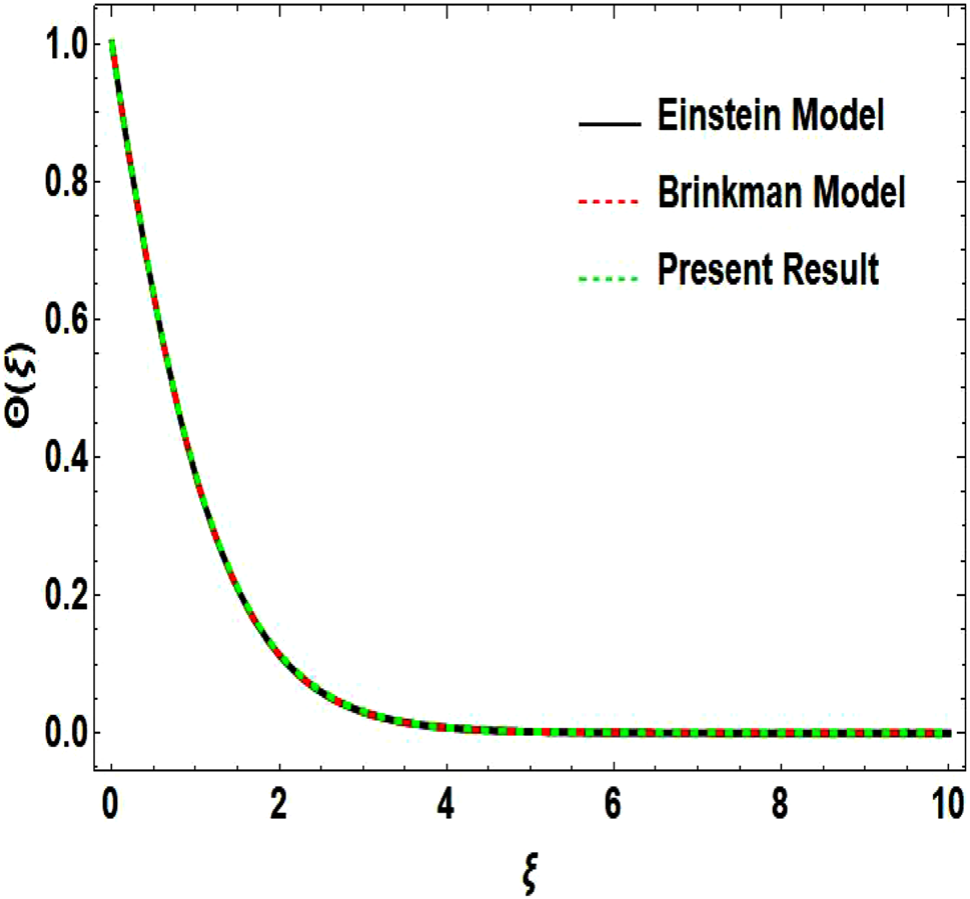
Temperature curve relative to different models.

**Figure 6:**
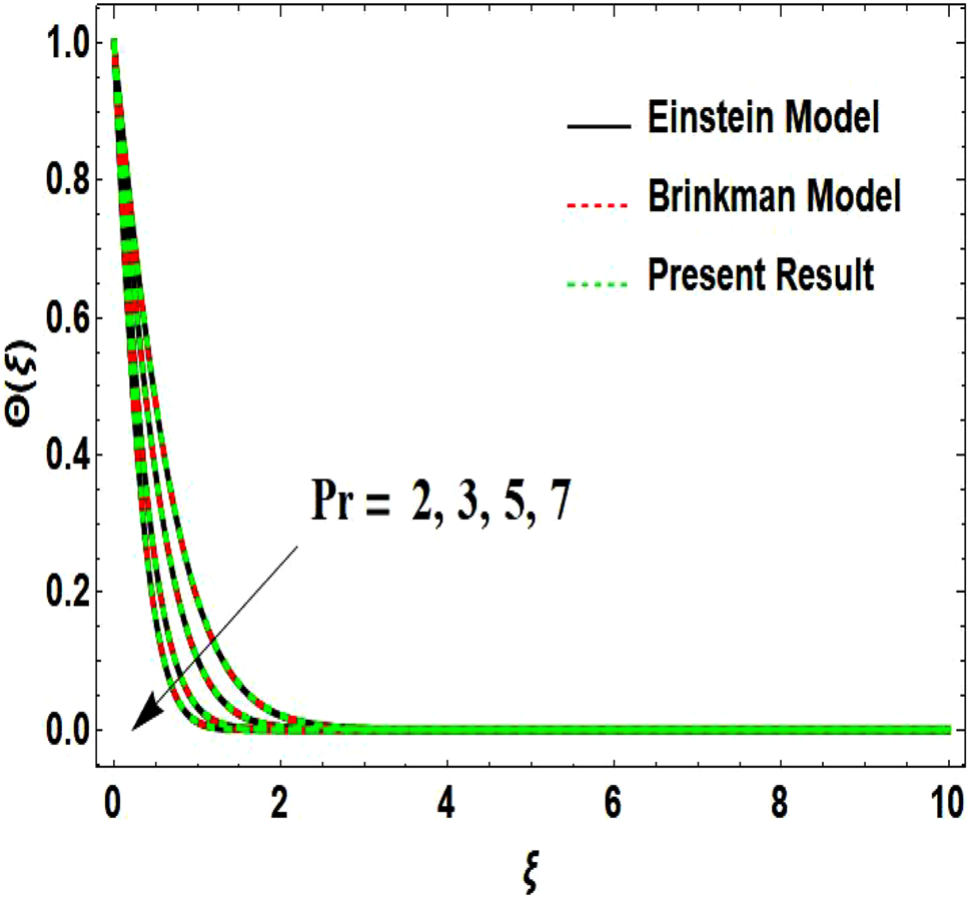
Temperature curve influenced by Prandtl number.

**Figure 7:**
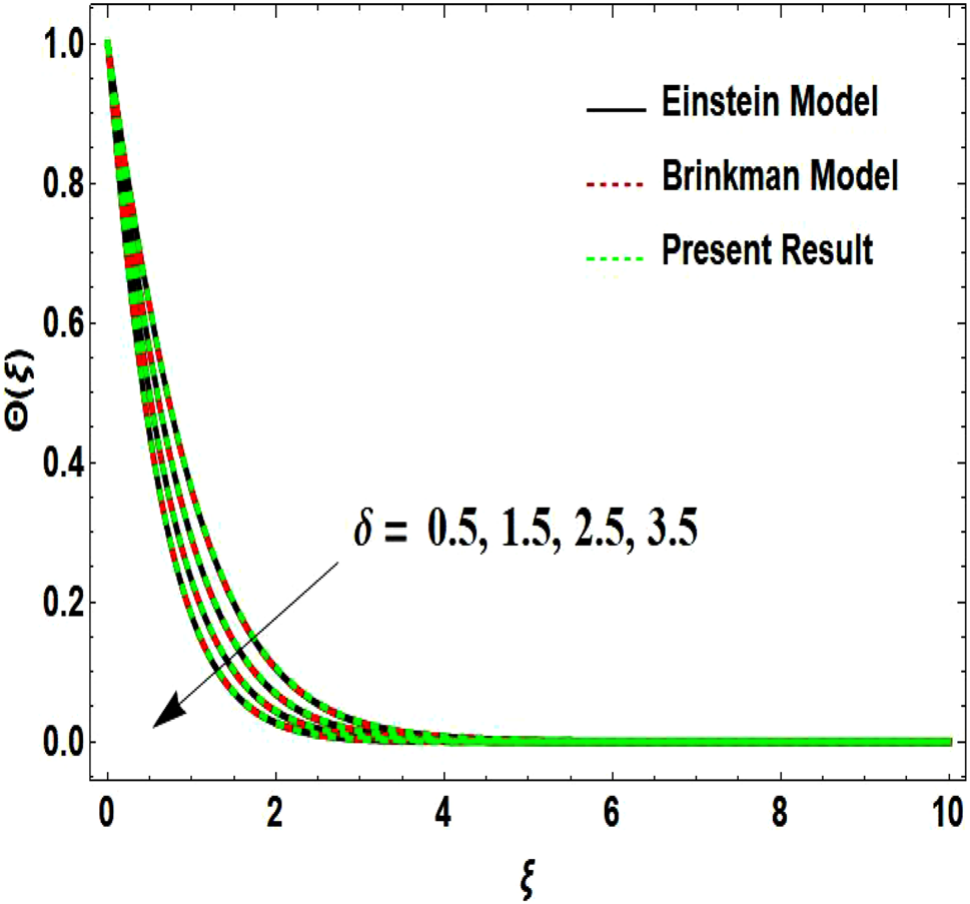
Temperature curve influenced by surface wall thickness parameter.

**Figure 8:**
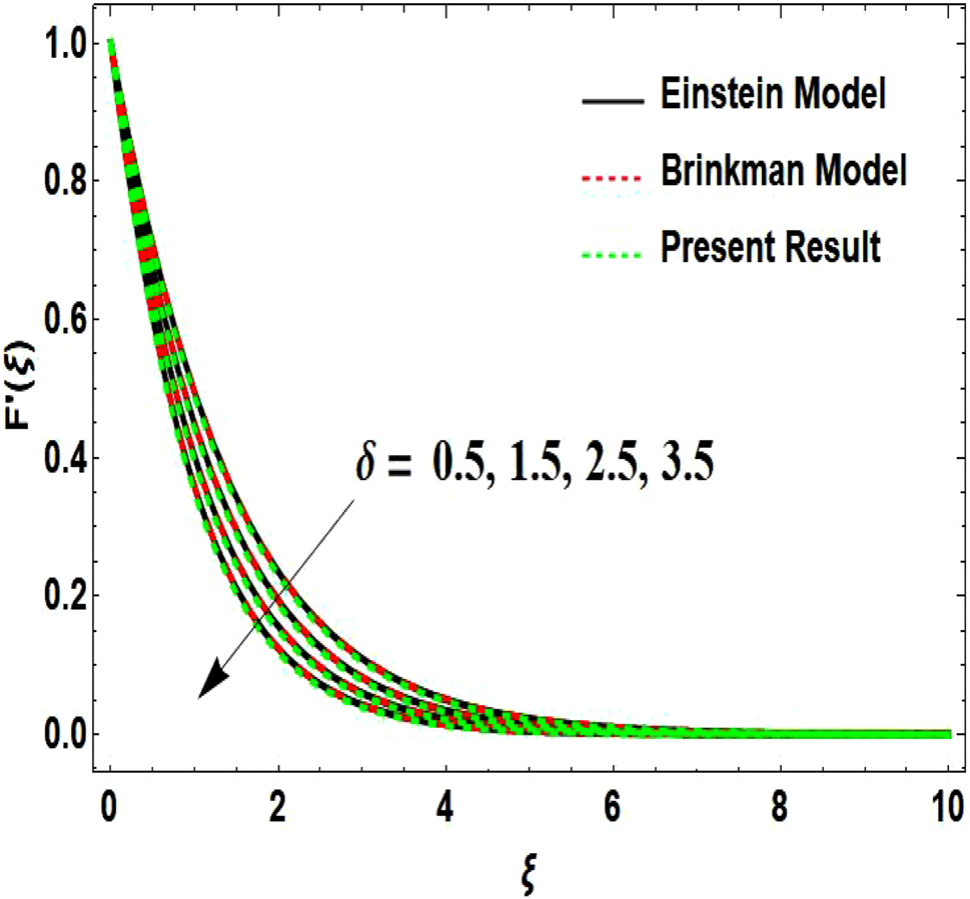
Velocity curve influenced by surface wall thickness parameter.

**Figure 9:**
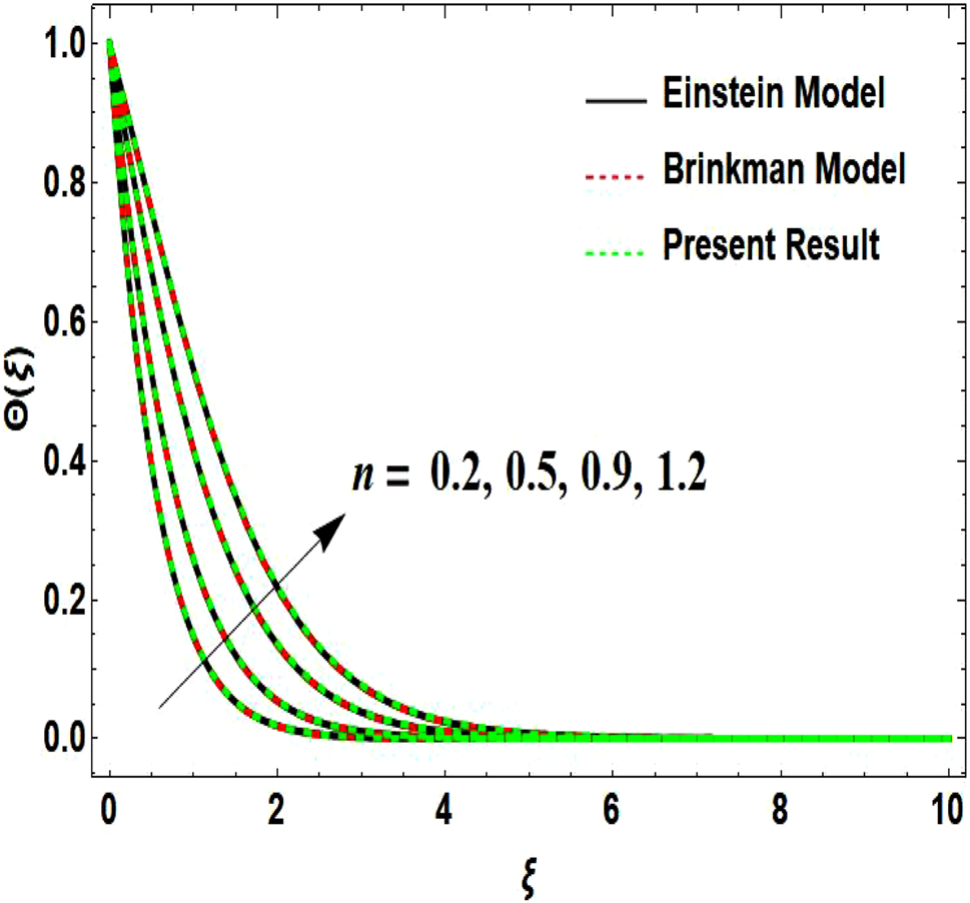
Temperature curve influenced by velocity power index parameter.

**Figure 10:**
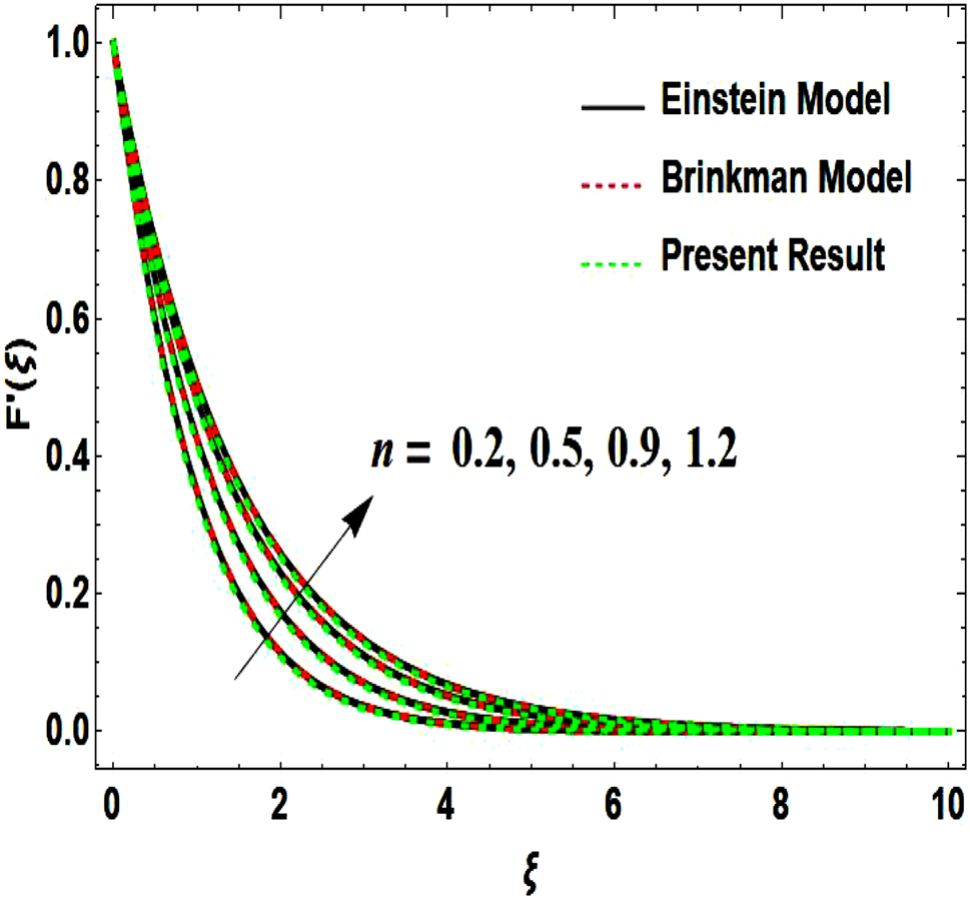
Velocity curve influenced by velocity power index parameter.

**Figure 11:**
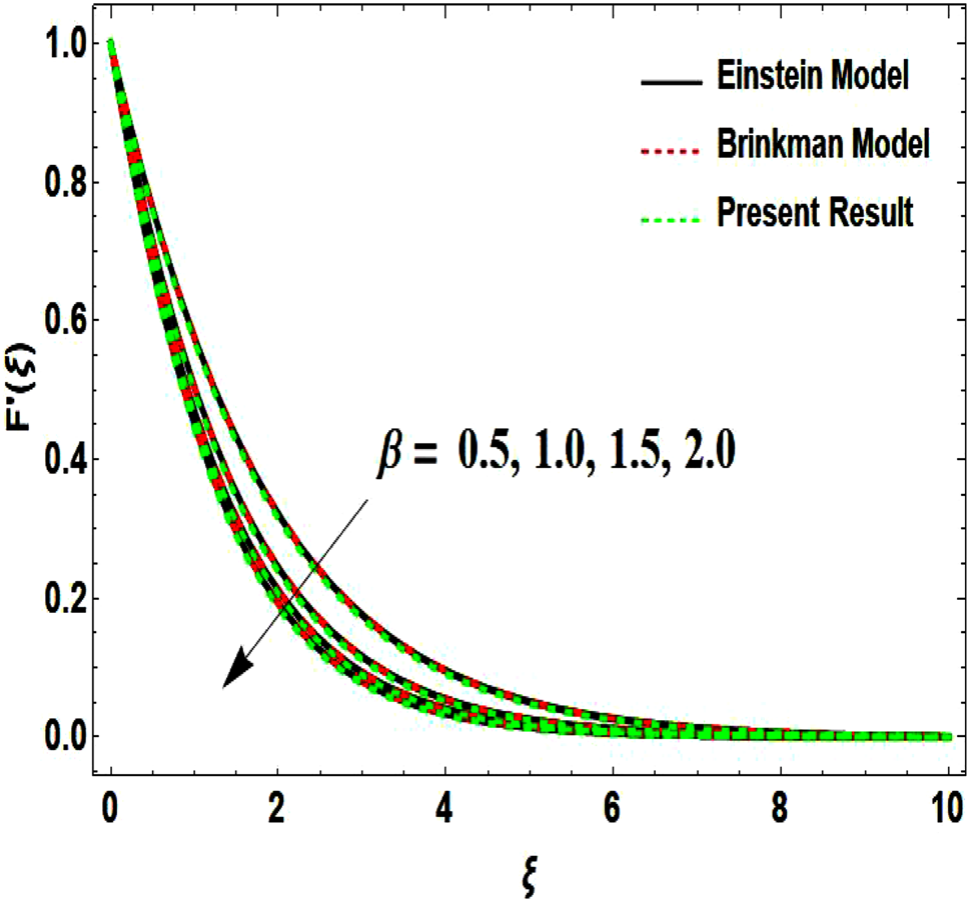
Velocity curve influenced by velocity power index parameter.

The two-dimensional flow behavior of non-Newtonian Casson fluid with water-based ${\mathrm{Al}}_{2}{\mathrm{O}}_{3}$ nanoparticle is inspected in the current study. The nonlinear coupled Eqs. (12) and (13) with Eq. (14) are worked out through the symbolic package Mathematica. In the ongoing analysis, we take the exponential form of the nanofluid viscosity. In using the nanofluid viscosity of exponential form, we can take different positive values of the variable $A$. To validate this point, we draw the temperature and velocity curves in Figs. 2 and 3 by taking different values of constant $A$. These figures demonstrate that the temperature and velocity profiles exhibit decreasing and increasing behavior respectively with various positive values of the constant $A$. The Einstein model, Brinkman model, and exponential expression of nanofluid viscosity $(A=1)$ are utilized to discuss the features of the flow problem such as temperature and velocity distributions. For the validation of these three models of viscosity to the ongoing flow problem, the velocity and temperature distributions relative to these models are manifested in Figs. 4 and 5. These figures present the validation of all three models since the boundary conditions of the velocity and temperature are satisfied. In Table 2, the expressions of different viscosity models are included to compute the values of surface drag force ${C}_{f}{{Re}_{x}}^\frac{1}{2}$ and heat transfer rate ${Nu}_{x}{{Re}_{x}}^{-\frac{1}{2}}$. In all three models of nanofluid viscosity, the physical quantities exhibit approximated values relative to the pertinent parameters. The numerical values of the skin friction coefficient and Nusselt number decrease with the velocity power index parameter in all three models of viscosity. The surface thickness parameter enhances the heat transfer rate but decreases the skin friction coefficient corresponding to the three models. These numerical values and graphical illustrations in Figs. 4 and 5 show that the three different models of nanofluid viscosity are valid for the current flow problem. The significance of the pertinent parameters on various aspects of the system relative to the three different models of the nanofluid viscosity is graphically explained. Figure 6 is sketched to scrutinize the field of the temperature relative to the greater magnitude of the Prandtl number. It is contemplated that the higher amount of the Prandtl number in all three cases of viscosity, lowers the temperature curve. Physically, the ratio of momentum diffusivity and thermal diffusivity is defined by the Prandtl number. As thermal diffusivity is the measure of a material’s capability to diffuse heat. The amount of thermal diffusivity becomes lowers due to the larger Prandtl number which means that the heat transfer rate is minimum in the fluid. Accordingly, the temperature distribution exhibits a declining nature. The decreasing nature of the fields of temperature and fluid velocity is outlined in Figs. 7 and 8 correspond to the larger values of the surface thickness parameter. The reason is that the higher amount of the surface thickness parameter extends the thickness of the concerned surface. The process of the heat transfer to fluid from the sheet surface reduces as the heat rapidly transfers through a thinner surface as compared to a thicker surface. As a result, there is a decline in the curve of the temperature distribution. Moreover, the stretched surface velocity diminishes with the escalating thickness of the surface which minimizes the flow velocity. Figures 9 and 10 are prepared to indicate the influence of the velocity power index parameter on the curve of the temperature distribution and flow velocity respectively. Both the temperature and velocity profiles exhibit an accelerating behavior. Physically, the slendering surface becomes thinner with the greater intensity of the velocity index parameter. As earlier discussed, through the thinner surface, the transfer of heat becomes excessive which correspondingly accelerates the temperature field. Likewise, the velocity power index parameter has a direct relation with the surface velocity. As a result, the increasing stretched velocity escalates the flow velocity within the boundary layer region. Figure 11 exhibits the reduction in the profile of the fluid flow with the higher magnitude of the Casson fluid parameter. This behavior is true due to the fact that the fluid viscosity is increased with the greater intensity of the Casson fluid parameter and accordingly the fluid velocity decreases due to the larger viscosity.

**Table 2:** Comparison of the values of ${C}_{f}{{Re}_{x}}^\frac{1}{2}$ and ${Nu}_{x}{{Re}_{x}}^{-\frac{1}{2}}$ with distinct models of viscosity.

$\beta$	$n$	$\delta$	Einstein model^27^		Brinkman Model^28^		Present Result	
			${C}_{f}{{Re}_{x}}^\frac{1}{2}$	${Nu}_{x}{{Re}_{x}}^{-\frac{1}{2}}$	${C}_{f}{{Re}_{x}}^\frac{1}{2}$	${Nu}_{x}{{Re}_{x}}^{-\frac{1}{2}}$	${C}_{f}{{Re}_{x}}^\frac{1}{2}$	${Nu}_{x}{{Re}_{x}}^{-\frac{1}{2}}$
**0.5**	0.5	0.3	− 2.9138	0.79975	− 2.9162	0.79986	− 2.8734	0.79798
**1.0**			− 2.3959	0.77193	− 2.3979	0.77206	− 2.3630	0.76970
**1.5**			− 2.1956	0.75723	− 2.1974	0.75738	− 2.1656	0.75478
	**0.6**		− 3.0768	0.77462	− 3.0794	0.77473	− 3.0337	0.77281
	**0.9**		− 3.5203	0.70623	− 3.5233	0.70634	− 3.4700	0.70432
	**1.1**		− 3.7869	0.66531	− 3.7902	0.66542	− 3.7324	0.66335
		**0.5**	− 2.9799	0.83586	− 2.9823	0.83596	− 2.9395	0.83405
		**0.6**	− 3.0134	0.85422	− 3.0158	0.85433	− 3.0130	0.85241
		**0.7**	− 3.0472	0.87279	− 3.0496	0.87290	− 3.0069	0.87096

Significant values are in bold.

## Concluding remarks

An analysis of an unsteady flow phenomenon of a nano non-Newtonian Casson fluid over a slendering surface with inconsistent thickness is exhibited in this study. The combination of the pure water base fluid and nanoparticle Aluminum Oxide is utilized in the development of the concerned nanofluid. A new exponential type of nanofluid viscosity with Einstein and Brinkman models is implemented throughout the flow analysis. A graphical comparison is conducted between the nanofluid exponential viscosity, Einstein model, and Brinkman model for fluid flow and temperature field. The ongoing study exhibits the following main points.

- The flow velocity becomes diminishes by strengthening the Casson fluid parameter.
- The profile of the temperature presents declining behavior corresponding to the higher values of the Prandtl number.
- Both the flow velocity and temperature distribution are the decreasing functions of the surface thickness parameter.
- The accelerating magnitude of the velocity power index parameter enhances the behavior of the temperature and velocity distributions.
- In three different models of nanofluid viscosity, the features of the flow problem exhibit excellent behavior.
- There exists a good relationship between the values of heat transfer rate and surface drag force corresponding to the distinct models of viscosity.
- In the exponential model of the viscosity, the constant $A$ takes only the positive values for the current analysis.
- The current flow problem can also be analyzed for other non-Newtonian fluids with distinct nanoparticles. Various physical effects can be implemented on the ongoing flow problem.

## Data Availability

The authors states that all the files are provided in the paper no hidden file is required however if journal required any further data from us, we will provide and the corresponding author is responsible to provide to the journal.

## List of symbols

$\beta$: Casson fluid parameter
$$\widetilde{u},\widetilde{v}$$: Velocity elements
$${\widetilde{u}}_{0},a$$: Positive constants
$n$: Velocity power index parameter
$J$: Stretchable sheet coefficient
${\tau }_{w}$: Surface shear stress
$$x,y$$: Cartesian coordinates
$${\widetilde{T}}_{\infty }$$: Ambient temperature
$$\widetilde{s}$$: Nanoparticle
$\infty$: Free stream condition
$\delta$: Surface thickness parameter
${c}_{p}$: Specific heat capacity
$$\widetilde{f}$$: Base fluid
$$k$$: Thermal conductivity
$${\widetilde{T}}_{w}$$: Surface temperature
$$Pr$$: Prandtl number
$$\rho$$: Density
$$\upsilon$$: Kinematic viscosity
${Re}_{x}$: Local Reynolds number
${q}_{w}$: Surface heat flux
$\phi$: Volume fraction of nanoparticle
$$n\widetilde{f}$$: Nanofluid
